# Screening for autophagy/hypoxia/ferroptosis/pyroptosis-related genes of tendon injury and repair in a rat model after celecoxib and lactoferrin treatment

**DOI:** 10.1186/s13018-023-03856-9

**Published:** 2023-05-25

**Authors:** Yaonan Zhang, Lei Shi, Fei Wang, Lin Wang, Nan Min, Liangyuan Wen, Qingyun Xue

**Affiliations:** Orthopaedic Department, Beijing Hospital; National Center of Gerontology; Institute of Geriatric Medicine, Chinese Academy of Medical Sciences, Beijing, People’s Republic of China

**Keywords:** Tendon injury, Repair, Celecoxib, Lactoferrin

## Abstract

**Background:**

Tendon injuries are among the most common musculoskeletal disorders. Celecoxib possesses an effective anti-inflammatory activity in the tendon injury treatment. Lactoferrin has a great potential for the tendon regeneration. However, the efficacy of celecoxib combined with lactoferrin in the treatment of tendon injury has not been reported. In this study, we aimed to investigate the effect of celecoxib and lactoferrin on tendon injury and repair, and screen for the crucial genes associated with the tendon injury and repair.

**Methods:**

The rat tendon injury models were established and divided into four groups: normal control group (*n* = 10), tendon injury model group (*n* = 10), celecoxib treatment group (*n* = 10), and celecoxib + lactoferrin treatment group (*n* = 10). Then, RNA sequencing was performed to identify differentially expressed lncRNAs (DElncRNAs), miRNAs (DEmiRNAs) and mRNAs (DEmRNAs) in celecoxib treatment group and celecoxib + lactoferrin treatment group. Next, autophagy/hypoxia/ferroptosis/pyroptosis-related DEmRNAs were further identified. Subsequently, functional enrichment, protein–protein interaction (PPI) network and transcriptional regulatory network construction for these genes were performed.

**Results:**

The animal study demonstrated that combinational administration of celecoxib with lactoferrin rescued the harmful effects caused by celecoxib in the treatment of tendon injury. Compared to tendon injury model group, 945 DEmRNAs, 7 DEmiRNAs and 34 DElncRNAs were obtained in celecoxib treatment group, and 493 DEmRNAs, 8 DEmiRNAs and 21 DElncRNAs were obtained in celecoxib + lactoferrin treatment group, respectively. Subsequently, 376 celecoxib + lactoferrin treatment group-specific DEmRNAs were determined. Then, 25 DEmRNAs associated with autophagy/hypoxia/ferroptosis/pyroptosis were identified.

**Conclusions:**

Several genes, such as, Ppp1r15a, Ddit4, Fos, Casp3, Tgfb3, Hspb1 and Hspa8, were identified to be associated with tendon injury and repair.

**Supplementary Information:**

The online version contains supplementary material available at 10.1186/s13018-023-03856-9.

## Introduction

Musculoskeletal disorders exert the significant detrimental effect on the life quality of patients [1]. Multiple pathological factors are associated with tendon injury, including trauma, aging, inflammation, chronic overuse, and genetic factors. The mechanisms of injury include some types of overload or overuse of the tendon that most likely lead to tendon degeneration, cell phenotype changes and hypervascularization [2]. In the clinical work, nonsteroidal anti-inflammatory drugs (NSAIDs) are used to treat tendon injury, such as, selective COX-2 inhibitor and celecoxib. Celecoxib possesses an effective analgesic and anti-inflammatory activity in the tendon injury treatment [3]. The side effects of celecoxib include inhibition of tendon cell proliferation and migration, which can adversely affect tendon healing [4]. Lactoferrin, an iron-binding glycoprotein, functions as an osteogenic growth factor and enhance the proliferation and differentiation of osteoblasts, and has a great potential for the tendon regeneration [5]. Moreover, when lactoferrin is used in combination with non-steroidal anti-inflammatory drugs, the inhibitory effects on tenocyte proliferation, viability, and collagen formation are rescued [6]. We have previously shown that lactoferrin is anabolic to human tenocytes in vitro and reverses potential inhibitory effects of NSAIDs on human tenocytes [7]. To date, the efficacy of celecoxib combined with lactoferrin in the treatment of tendon injury has not been reported.

Tendon fibroblasts play an important role in remodeling phase of wound healing [8]. In tendon injury, high glucose can repress the proliferation of tendon fibroblasts by inhibiting autophagy activation [9]. It has been indicated that hypoxia has a critical function in chondrogenesis, osteogenesis and angiogenesis, and plays an essential role in the tissue repair process [10]. Zhao et al. reported that hypoxia was essential for bone–tendon junction healing [11]. Yu et al. suggested that hypoxia enhanced tenocyte differentiation of adipose-derived mesenchymal stem cells [12]. Chen et al. demonstrated that hypoxia-induced mesenchymal stem cells exhibited stronger tenogenic differentiation capacities [13]. Ferroptosis, a new form of regulated cell death (driven by iron-dependent lipid peroxidation), is involved in a variety of diseases [14]. Iron is a key element that plays a crucial role in mammalian cells (such as osteoclast) [15]. Ni et al. demonstrated that ferroptosis was involved in osteoclastogenesis [16]. Pyroptosis (also known as cell inflammatory necrosis) is a programmed cell death mode closely associated with the inflammatory response and mediated by caspase-1 or caspase-11 [17, 18]. In alveolar bone, high glucose concentration may activate pyroptosis to inhibit the proliferation and differentiation of osteoblasts [19]. Under certain pathological conditions, pyroptosis may occur in osteoblasts, affect their proliferation and differentiation and consequently affect the development and morphological changes of bone tissue [20].

In this study, we performed animal studies to investigate the efficacy of celecoxib combined with lactoferrin in the treatment of tendon injury. Then, differentially expressed mRNAs (DEmRNAs), differentially expressed miRNAs (DEmiRNAs) and differentially expressed lncRNAs (DElncRNAs) in celecoxib treatment group and celecoxib + lactoferrin treatment group were identified. As we mentioned above, autophagy/hypoxia/ferroptosis/pyroptosis may be associated with tendon injury and repair. Hence, autophagy/hypoxia/ferroptosis/pyroptosis-related gene sets were downloaded for subsequent analysis. In this study, we aimed to investigate the effect of celecoxib and lactoferrin on tendon injury and repair, and screen for the crucial genes associated with the tendon injury and repair.

## Materials and methods

### Animal model

A total of 40 six-week-old Sprague–Dawley rats weighting 200–300 g were used. The rats were housed under a 12-h light/dark cycle in a pathogen-free area with free access to water and food. All animals were treated according to institutional guidelines for laboratory animal treatment and care. All experimental procedures were approved by the Animal Research Ethics Committee of our hospital.

Rats were randomly divided into four groups: normal control group (*n* = 10), tendon injury model group (*n* = 10), celecoxib treatment group (*n* = 10), and celecoxib + lactoferrin treatment group (*n* = 10). Lactoferrin was purchased from Wuhan Nuohui Pharmaceutical & Chemical Co., LTD. Following 1 week of feeding and adaptation, the rats were anesthetized. The midpoint of the achilles tendon was transected and sutured immediately. The tendon was not cut in the normal control group, and other operations were the same as those in the experimental group. Celecoxib (10 mg/kg) were injected daily at the site of injury in each rat in celecoxib treatment group. Celecoxib (10 mg/kg) and lactoferrin (2 g/kg) were injected daily at the site of injury in each rat in celecoxib and lactoferrin treatment group.

### Hematoxylin and eosin (HE) staining, Masson staining and immunohistochemistry

Rats were sacrificed on the 1st, 14th, and 28th day after surgery. Three rats in each group were sacrificed on the 1st and 14th day, and four rats were sacrificed on the 28th day. Then, the tendon of the injured site was collected. For histological analysis, tendons were fixed in 4% paraformaldehyde for 24 h. Sagittal paraffin sections were prepared by embedding the samples in paraffin and cutting into 4 µm thick sections. Four sections per sample were stained with HE and observed under the OLYMPUS EX-51 microscope (Tokyo, Japan) at 100 × magnification. Masson’s trichrome stain (Solarbio, G1340) was performed according to kit directions. RECA-1 is a cell surface antigen expressed by rat endothelial cells. In this study, RECA-1 was selected for immunohistochemistry analysis on days 1, 14 and 28. Then, 5-μm-thick continuous sections were incubated with an anti-RECA-1 antibody (abcam, ab22492, 1:1000) produced in rabbit followed by goat anti-rabbit immunoglobulin antibody conjugated by horseradish peroxidase. Then, slides were visualized using diaminobenzidine (DAB) substrate.

### RNA isolation and sequencing

We selected samples from injury model group, celecoxib treatment group and celecoxib + lactoferrin treatment group on day 14 for subsequent RNA sequencing. Total RNA was harvested using TRIzol reagent (Invitrogen, Carlsbad, CA, USA) from the tendons. With Agilent 2100 and Nanodrop2000, the quality of RNA was assessed. The quality of the libraries was determined using an Agilent 2100 Bioanalyzer and ABI StepOnePlus Real-Time PCR System. Illumina Hiseq x-ten platform was used to perform RNA sequencing for mRNA and lncRNA. The raw sequencing data were submitted to sequencing quality control by FastQC. Reads with low quality were removed. BGISEQ-500 platform was used for RNA sequencing for miRNA. The Fastx-Toolkit was used to trim 5’ and 3’ segments of reads to remove bases (with mass < 20 and delete reads with N > 10%). The Rfam was used for annotation analysis on measured small RNA with BLAST v2.3.0. The mature miRNA and miRNA precursor sequences were downloaded from miRBase. The expression of miRNA was quantified with miRDeep2. HISAT2 was used to align the clean reads with the reference genome, Ensemble Rnor_6.0.

### Differential expression analysis

By using DESeq2, the DEmRNAs and DElncRNAs were identified with *p*-value < 0.05. With DEGseq2, DEmiRNAs were identified with *p*-value < 0.05 as well. With R package “pheatmap,” hierarchical clustering analysis was performed. David 6.8 was applied to perform GO and KEGG enrichment analysis for DEmRNAs with *p*-value < 0.05. Particularly, celecoxib + lactoferrin treatment group-specific DEmRNAs were further obtained.

### Identification of genes associated with autophagy/hypoxia/ferroptosis/pyroptosis

Autophagy-related genes were extracted from Human Autophagy Database (HADb, http://www.autophagy.lu/index.html) and the GOBP REGULATION OF AUTOPHAGY gene set in Molecular Signatures Database (MSigDB). Totally, 516 autophagy-related genes were included for subsequent analysis. Then, 200 hypoxia-related genes were retrieved from the MSigDB. A total of 267 ferroptosis-related genes were retrieved from the FerrDb dataset (http://www.zhounan.org/ferrdb/) and the previous literature [21]. In addition, 41 pyroptosis-related genes were retrieved from the previous literature [22–24]. Finally, genes associated with autophagy/hypoxia/ferroptosis/pyroptosis were obtained by overlapping celecoxib + lactoferrin treatment group-specific DEmRNAs with autophagy/hypoxia/ferroptosis/pyroptosis-related genes, respectively.

### Functional annotation and protein–protein interaction (PPI) network construction

In order to explore the biological functions and the potential pathways of the genes associated with autophagy/hypoxia/ferroptosis/pyroptosis, David 6.8 was utilized to perform GO and KEGG enrichment analysis. A *p*-value < 0.05 was considered statistically significant. Online database STRING (https://string-db.org) was used to analyze the PPI networks.

### Construction of transcriptional regulatory networks

Potential transcription factors (TFs) targeted to the genes associated with autophagy/hypoxia/ferroptosis/pyroptosis were identified using the transcriptional regulatory relationships unraveled database. The TF-gene regulatory networks were visualized by using Cytoscape software.

## Results

### Celecoxib combined with lactoferrin treatment decreased inflammatory cell and the degree of fiber structure disorder

**Fig. 1:**
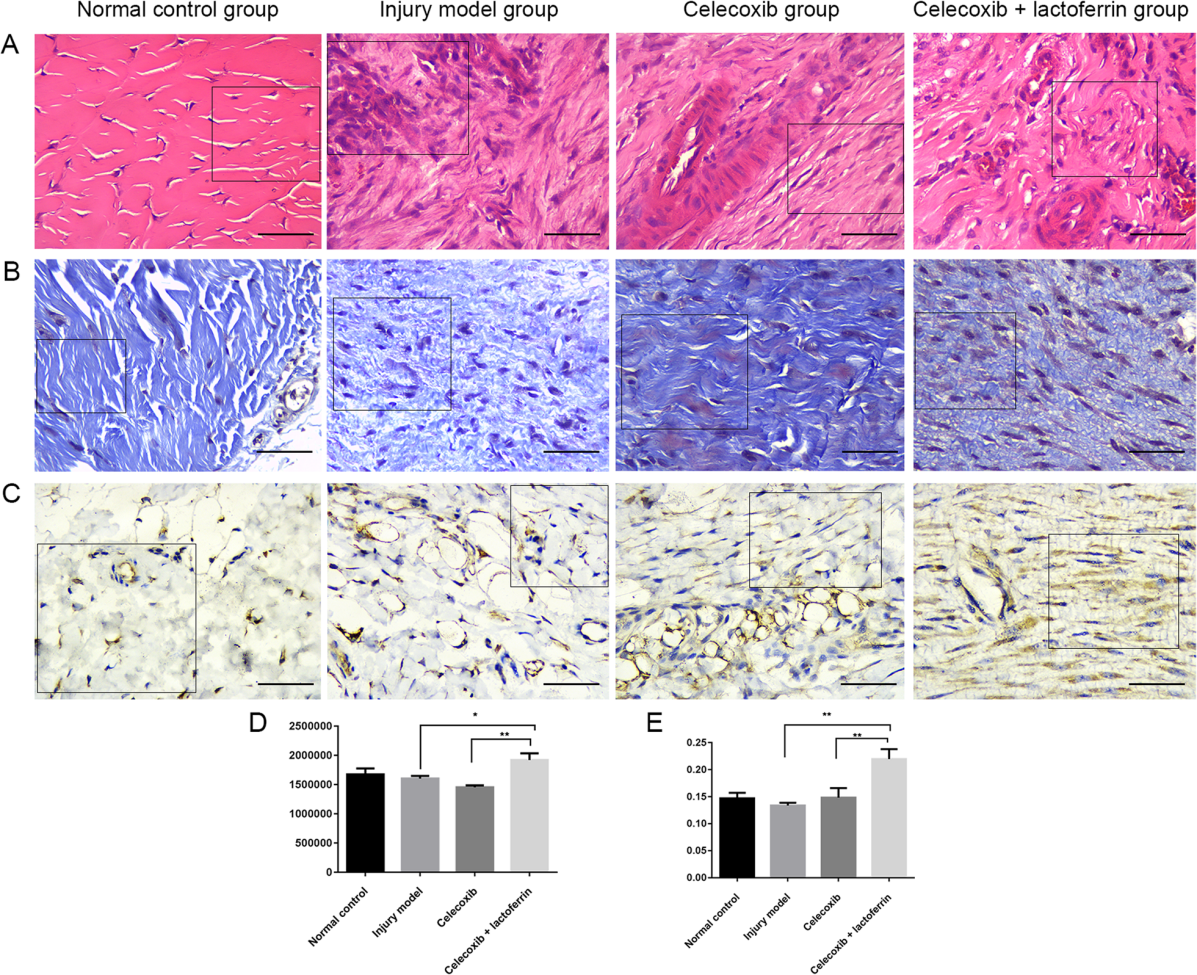
HE staining (**A**), Masson staining (**B**) and immunohistochemistry (**C**) of tendons in normal control group, injury model group, celecoxib treatment group, celecoxib + lactoferrin treatment group on day 14. Scale bar = 50 μm. **D** Quantification analysis of Masson staining. **E** Quantification analysis of immunohistochemistry. * indicates *p* < 0.05, ** indicates *p* < 0.01

In Fig. 1A, HE staining confirmed that the injured tendon had more inflammatory cell nuclei, obvious inflammation infiltration, and disorder of fiber structure in injury model group compared with the other three groups. The number of inflammatory cell nuclei decreased in the celecoxib treatment group and celecoxib + lactoferrin treatment group compared with the injury model group. The degree of fiber structure disorder also decreased in the celecoxib treatment group and celecoxib + lactoferrin group compared with the injury model group. In Fig. 1B and 1D, Masson’s trichrome staining showed that the injury model group had fewer collagen fibers and the collagen fiber structure was obviously disordered compared with the normal control group. Compared with the celecoxib treatment group, celecoxib + lactoferrin group formed new collagen fibers and increased the proportion of positive staining area. In Fig. 1C and 1F, compared with the injury model group, the distribution of RECA-1 protein in the celecoxib + lactoferrin group significantly increased. The distribution range of RECA-1 protein in the celecoxib treatment group and celecoxib + lactoferrin group increased, and the color became darker, indicating that the inflammation recovery period led to the proliferation of new capillaries and increased blood vessels. According to the scope and color depth, the celecoxib + lactoferrin treatment group has a wider range and darker color than the celecoxib treatment group, indicating that the inflammatory recovery is better in celecoxib + lactoferrin treatment group compared with the celecoxib treatment group.

### Screening of DEmRNAs, DEmiRNAs and DElncRNAs

**Fig. 2:**
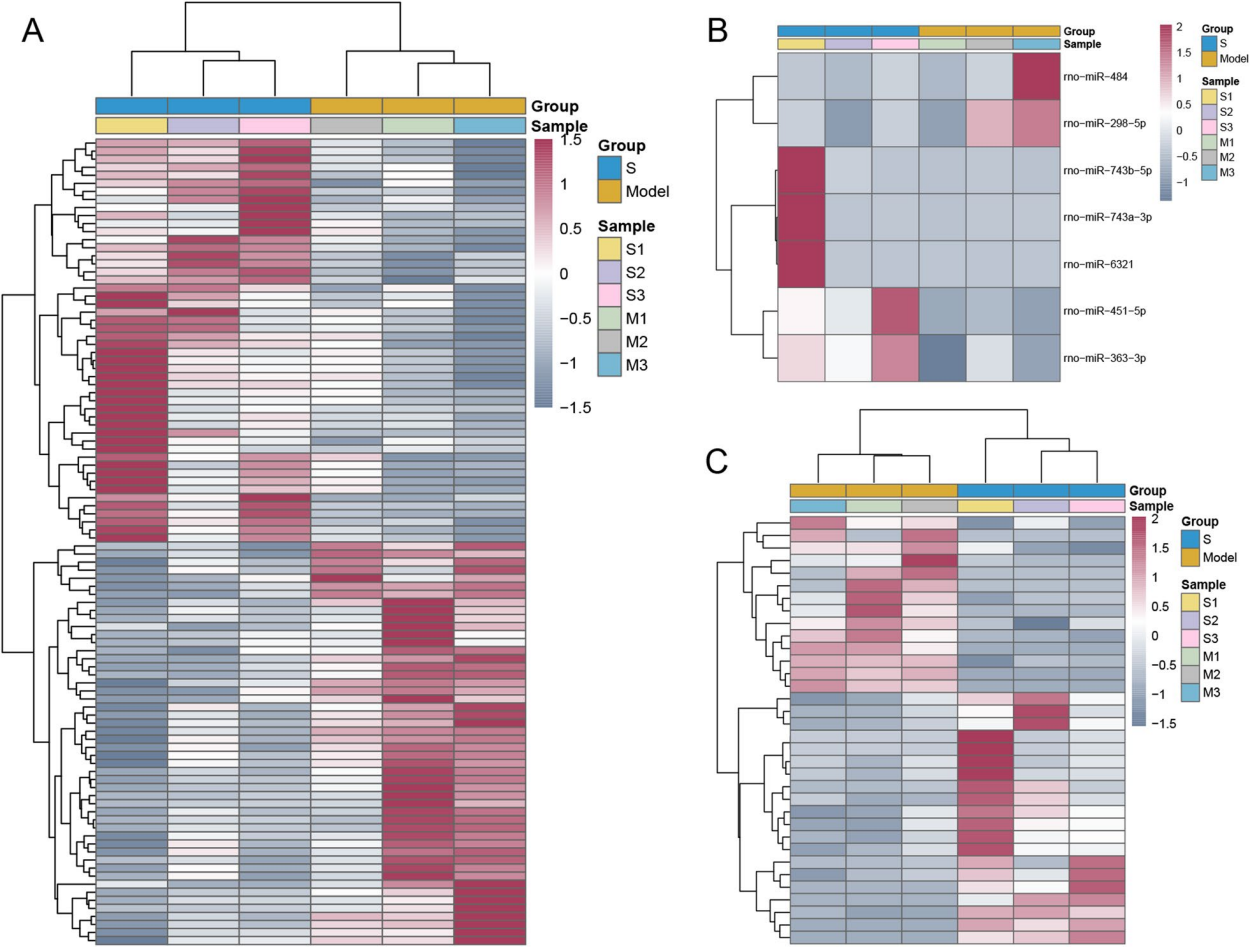
The heatmap of DEmRNAs (**A**), DEmiRNAs (**B**) and DElncRNAs (**C**) between injury model group and celecoxib treatment group on day 14

**Fig. 3:**
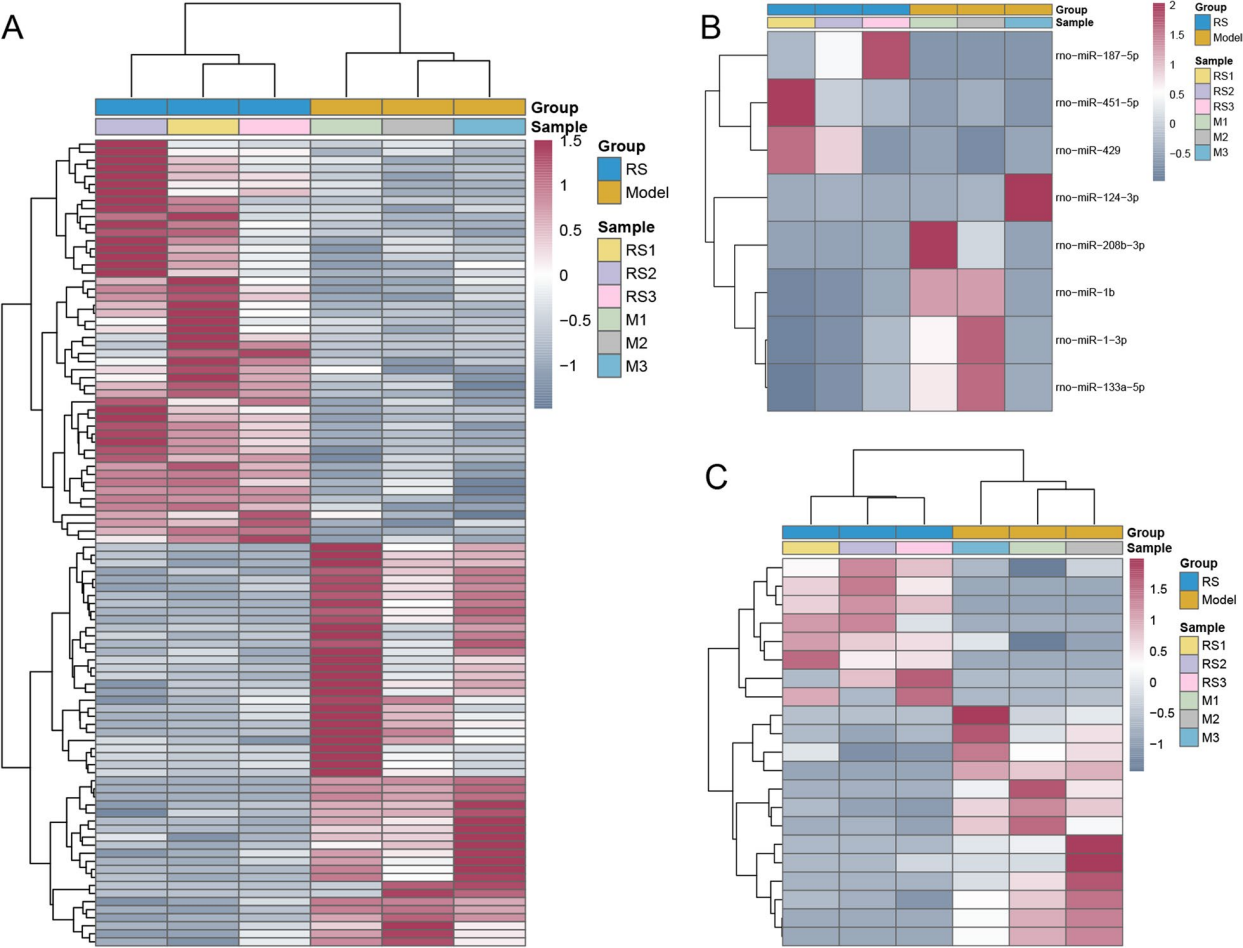
The heatmap of DEmRNAs (**A**), DEmiRNAs (**B**) and DElncRNAs (**C**) between injury model group and celecoxib + lactoferrin treatment group on day 14

Compared to tendon injury model group, 945 (548 up-regulated and 397 down-regulated) DEmRNAs, 7 (5 up-regulated and 2 down-regulated) DEmiRNAs and 34 (20 up-regulated and 14 down-regulated) DElncRNAs were obtained in celecoxib treatment group, and 493 (219 up-regulated and 274 down-regulated) DEmRNAs, 8 (3 up-regulated and 5 down-regulated) DEmiRNAs and 21 (8 up-regulated and 13 down-regulated) DElncRNAs were obtained in celecoxib + lactoferrin treatment group, respectively. The heatmap of DEmRNAs, DEmiRNAs and DElncRNAs is shown in Figs. 2 and 3. GO enrichment analysis revealed that these biological processes such as blood vessel development, bone development, muscle cell differentiation, and muscle tissue development were dysregulated in celecoxib treatment group (Additional file 1: Figure S1A). KEGG pathway analysis highlighted that MAPK signaling pathway, ECM-receptor interaction, T cell receptor signaling pathway and GnRH signaling pathway were dysregulated in celecoxib treatment group (Additional file 1: Figure S1B). GO enrichment analysis revealed that these biological processes such as skeletal muscle cell differentiation, response to hypoxia, positive regulation of smooth muscle cell proliferation and negative regulation of cell proliferation were dysregulated in celecoxib + lactoferrin treatment group (Additional file 2: Figure S2A). KEGG pathway analysis highlighted that MAPK signaling pathway, HIF-1 signaling pathway, cAMP signaling pathway and PI3K-Akt signaling pathway were dysregulated in celecoxib + lactoferrin treatment group (Additional file 2: Figure S2B). Due to the small number of DEmiRNAs and DElncRNAs, our next research focus is mainly on DEmRNAs. Subsequently, 376 (169 up-regulated and 207 down-regulated) celecoxib + lactoferrin treatment group-specific DEmRNAs were determined (Additional file 3: Figure S3). Also, the ceRNA network was built, but no results were available.

### Identification of genes associated with autophagy/hypoxia/ferroptosis/pyroptosis

In order to further investigate what role the celecoxib + lactoferrin treatment group-specific DEmRNAs play in tendon injury repair, the autophagy/hypoxia/ferroptosis/pyroptosis-related gene sets were retrieved from the corresponding databases and related literature. In total, 25 genes associated with autophagy/hypoxia/ferroptosis/pyroptosis were obtained by overlapping celecoxib + lactoferrin treatment group-specific DEmRNAs with autophagy/hypoxia/ferroptosis/pyroptosis-related genes, respectively (Table 1). In addition, 6 genes (Hspb1, Fos, Gapdh, Ppp1r15a, Casp3, and Ddit4) were associated with at least two of autophagy, hypoxia, ferroptosis, and pyroptosis.

**Table 1:** The list of autophagy/hypoxia/ferroptosis/pyroptosis-related genes

Gene	Type
HSPB1	Autophagy/ferroptosis-related gene
FOS	Autophagy/hypoxia-related gene
GAPDH	Autophagy/hypoxia-related gene
PPP1R15A	Autophagy/hypoxia-related gene
CASP3	Autophagy/pyroptosis-related gene
AMBRA1	Autophagy-related gene
EIF2AK2	Autophagy-related gene
HSPA8	Autophagy-related gene
IRGM	Autophagy-related gene
ITGB4	Autophagy-related gene
BMF	Autophagy-related gene
DNM1L	Autophagy-related gene
EIF4E	Autophagy-related gene
LRSAM1	Autophagy-related gene
NRBP2	Autophagy-related gene
PLEKHF1	Autophagy-related gene
TECPR1	Autophagy-related gene
DDIT4	Hypoxia/ferroptosis-related gene
SLC40A1	Ferroptosis-related gene
TFRC	Ferroptosis-related gene
CRYAB	Ferroptosis-related gene
CITED2	hypoxia-related gene
NOCT	Hypoxia-related gene
SLC6A6	Hypoxia-related gene
TGFB3	Hypoxia-related gene

### Functional annotation and PPI network construction

**Fig. 4:**
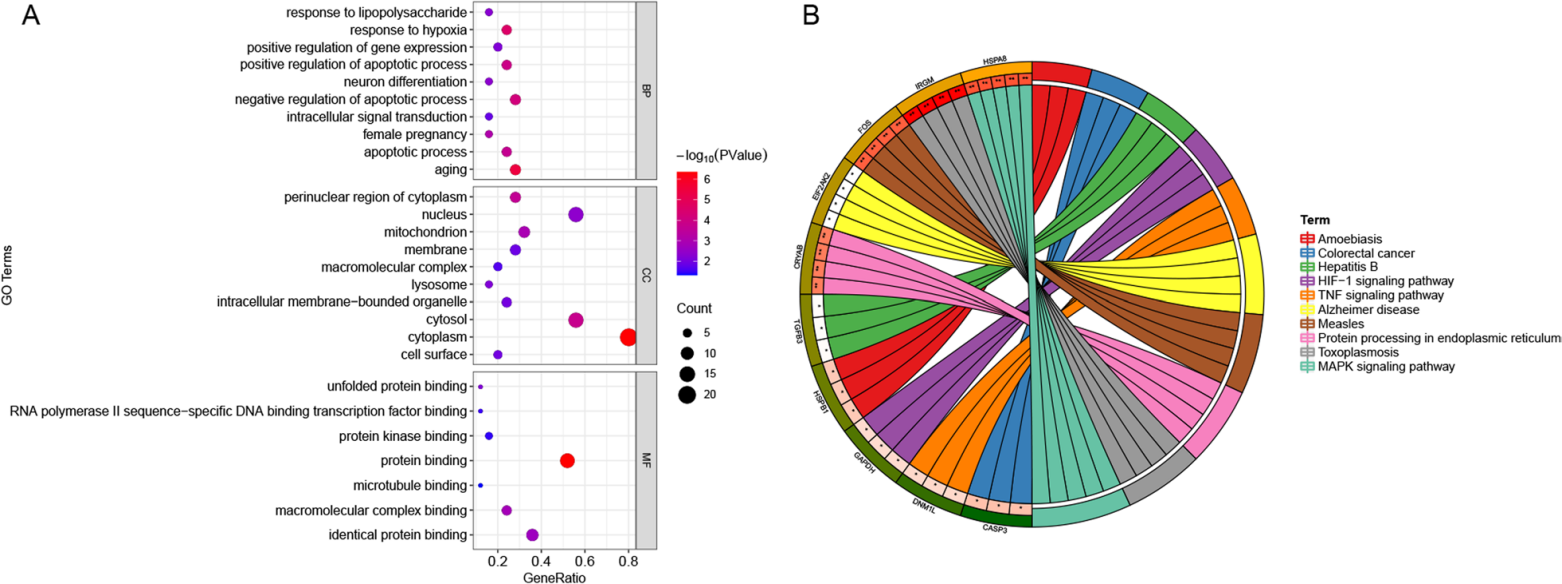
GO (**A**) and KEGG (**B**) enrichment analysis of the genes associated with autophagy/hypoxia/ferroptosis/pyroptosis BP: biological process; CC: cytological component; MF: molecular function

**Fig. 5:**
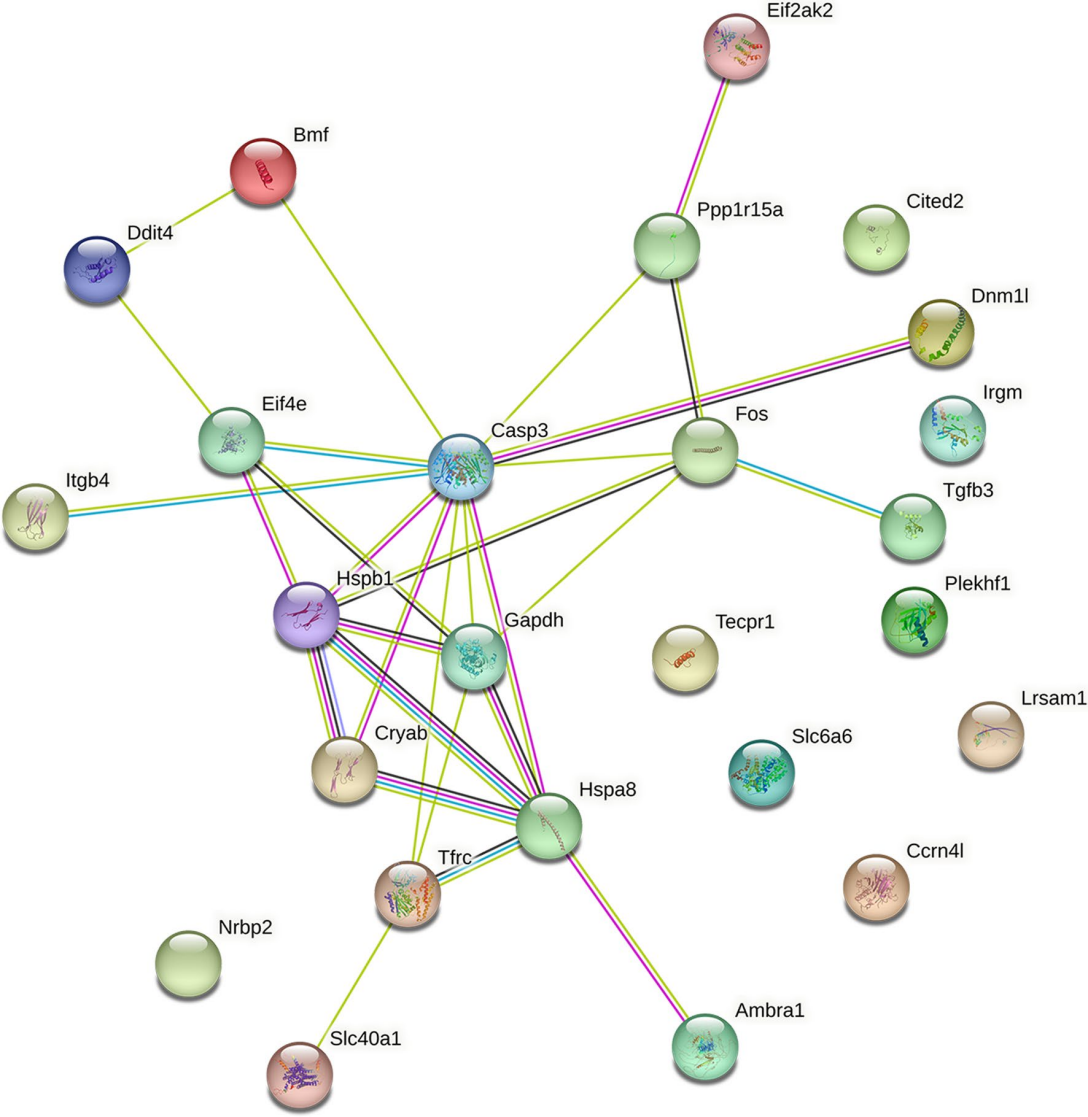
PPI network each node represents a protein, while each edge represents one protein–protein association

GO enrichment analysis revealed that these 25 genes were significantly enriched in negative regulation of apoptotic process, aging and response to hypoxia (Fig. 4A). KEGG pathway analysis highlighted that MAPK signaling pathway, HIF-1 signaling pathway and TNF signaling pathway were dysregulated (Fig. 4B). The PPI network included 25 proteins, which consisted of two subgroups: 17 proteins with strong connections with others and 8 separated proteins (Fig. 5). Among them, Hspa8 and Hspb1 had the highest interaction score, which was 0.951.

### Construction of TF-gene regulatory network

**Fig. 6:**
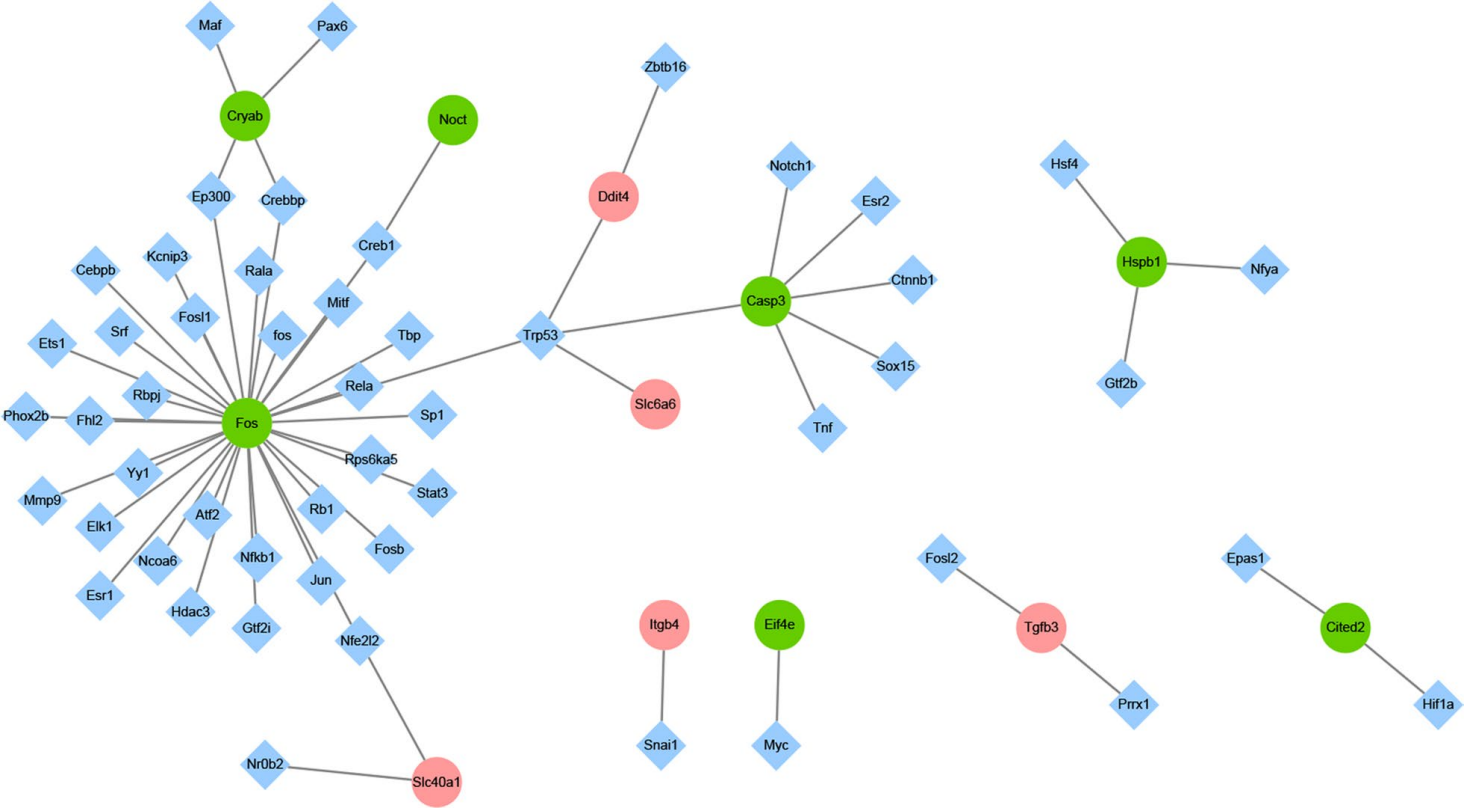
TF regulatory networks Red and green circles represent up- and down-regulated DEmRNAs. Rhombuses represent transcription factors (TFs). Edges indicate TF-DEmRNAs interactions

A total of 12 genes targeted by TFs were identified, including Casp3, Cited2, Cryab, Ddit4, Eif4e, Fos, Hspb1, Itgb4, Noct, Slc6a6, Slc40a1, and Tgfb3 (Fig. 6). Among which, Fos, Casp3, Slc6a6, Ddit4 can be regulated by Trp53. It is noted that Fos can be regulated by itself.

## Discussion

In this study, the rat tendon injury models were established and divided into four groups: normal control, tendon injury model, celecoxib treatment and celecoxib + lactoferrin treatment groups. Based on the results of HE staining, Masson staining and immunohistochemistry, we speculated that combinational administration of celecoxib with lactoferrin could rescue the harmful effects caused by celecoxib in the treatment of tendon injury. Then, the RNA sequencing and bioinformatics analysis indicated that compared to tendon injury model group, 945 DEmRNAs, 7 DEmiRNAs and 34 DElncRNAs were obtained in celecoxib treatment group, and 493 DEmRNAs, 8 DEmiRNAs and 21 DElncRNAs were obtained in celecoxib + lactoferrin treatment group. Given the small number DEmiRNAs and DElncRNAs obtained, we focused on DEmRNAs in the subsequent analysis. Then, 376 celecoxib + lactoferrin treatment group-specific DEmRNAs were determined. Next, 25 DEmRNAs associated with autophagy/hypoxia/ferroptosis/pyroptosis were obtained. Finally, several genes, such as, Ppp1r15a, Ddit4, Fos, Casp3, Tgfb3, Hspb1 and Hspa8, were identified to be associated with tendon injury and repair.

Aberrant mammalian target of rapamycin (mTOR) complex 1 (mTORC1) signaling is associated with altered bone homeostasis [25]. Protein phosphatase 1 regulatory subunit 15A (PPP1R15A), also known as GADD34, is a positive regulator of osteoclastogenesis and suppresses mTORC1 activity at the later stages of osteoclastogenesis [26]. DNA-damage-inducible transcript 4 (DDIT4) is an inhibitor of mTOR signaling [27]. DDIT4 confers a protective effect on radiation-induced premature senescence in osteoblast cells [28]. The JUN class of transcription factors is composed of heterdimers of Fos-related factors and Jun proteins, which is an important transcription factor in osteoblastic differentiation [29]. The c-Fos and c-Jun genes are the commonly studied member of the cellular immediate-early genes [30]. FOS is reported to improve tendon healing by promoting tendon cell proliferation and differentiation and regulating the inflammatory response [31]. A previous study suggested that ultrasound increased bone morphogenetic protein-2 expression in osteoblasts via the PI3K, Akt, c-Fos/c-Jun, and AP-1 signaling pathways [32].

Heat shock proteins are classified based on their molecular weights and include small HSPs, HSP40, HSP60, HSP70, HSP90, and large HSPs (HSP110 and glucoseregulated protein 170, GRP170) [33]. Heat shock protein family B (small) member 1 (HSPB1), also known as HSP27, is a member of the small heat shock protein family, which participates in the regulation of multiple physiological and pathophysiological cell functions. Elevated HSPB1 was related to estrogen-induced resistance to osteoblast apoptosis [34]. Unphosphorylated HSPB1 suppresses fibroblast growth factor-2-stimulated vascular endothelial growth factor release in osteoblasts [35]. HSPB1 suppressed platelet-derived growth factor-BB induced cell migration of osteoblasts [36]. Phosphorylated HSPB1 was involved in the pathogenesis of osteoporosis [37]. Heat shock protein family A (Hsp70) member 8 (HSPA8) is a member of the heat shock protein 70 family. The association of MNSFβ with HSPA8 may promote RANKL-induced osteoclastogenesis [38].

Caspase-3, one of the major activated cysteine proteases, constitutes the caspase family. You et al. investigated the effect of CASP3 inhibition on osteoblast differentiation capacity in high glucose conditions in mouse osteoblastic cell line MC3T3-E1 [39]. Mogi suggested that caspase activity could be required for osteogenic differentiation of osteoblastic cell [40]. Slawomir and his colleagues reported that CASP3, a pro-inflammatory factor involved in the TNF-α transduction pathway, was expressed in the injured tendon tissues of patients with rotator cuff tendinopathy [41]. CASP3 is suggested to be necessary for extracellular matrix remodeling in rat patellar tendon [42]. The transforming growth factor-β (TGFβ) family is expressed in various cell types and plays a crucial role in cellular division, migration, adhesion, differentiation, and programmed death [43]. The vascular smooth muscle cells proliferation pathways are known to be associated with various mRNAs and non-coding RNAs [44–46]. A certain level of active TGF-beta suppressed MMP-2 expression to promote the contractile phenotype of vascular smooth muscle cells [47]. TGF-β3 effectively protected against flexor tendon injury via regulating adhesion formation through the JNK/c-Jun pathway [48].

## Conclusions

In the present study, multiple bioinformatics analysis methods were applied to identify genes that play a key role in tendon injury and repair. Hspb1 was identified as an autophagy/ferroptosis-related gene, Fos and Ppp1r15a were identified as autophagy/hypoxia-related genes, Casp3 was identified as an autophagy/pyroptosis-related gene, Hspa8 was identified as an autophagy-related gene, Tgfb3 was identified as a hypoxia-related gene, and Ddit4 was identified as a hypoxia/ferroptosis-related gene. Hspa8 and Hspb1 had the highest interaction score in PPI network, which was 0.951. In addition, transcriptional regulatory network indicated that Fos can be regulated by itself. The above analysis results demonstrate the important role of these genes in tendon injury and repair from different perspectives. Also, the mechanism remained largely unknown. Our further research endeavors will concentrate on elucidating the fundamental mechanisms underlying the effect of celecoxib and lactoferrin on tendon injury and repair, including the intricate signaling pathways and protein expression profiles involved.

## Supplementary Information

**Additional file 1. Figure S1** GO (A) and KEGG (B) enrichment analysis of DEmRNAs between injury model and celecoxib treatment group BP: biological process; CC: cytological component; MF: molecular function.

**Additional file 2. Figure S2** GO (A) and KEGG (B) enrichment analysis of DEmRNAs between injury model group and celecoxib + lactoferrin treatment group BP: biological process; CC: cytological component; MF: molecular function.

**Additional file 3. Figure S3** Identification of celecoxib + lactoferrin treatment group-specific DEmRNAs (A) Venn diagram of up-regulated DEmRNAs in injury model group vs celecoxib treatment group and injury model group vs celecoxib + lactoferrin treatment group. (B) Venn diagram of down-regulated DEmRNAs in injury model group vs celecoxib treatment group and injury model group vs celecoxib + lactoferrin treatment group.

## Data Availability

The RNA-seq data have been deposited in the GSE201161 dataset (https://www.ncbi.nlm.nih.gov/geo/query/acc.cgi?acc=GSE201161).

## Abbreviations

DAB: Diaminobenzidine
DDIT4: DNA-damage-inducible transcript 4
DElncRNAs: Differentially expressed lncRNAs
DEmiRNAs: Differentially expressed miRNAs
DEmRNAs: Differentially expressed mRNAs
DUSP1: Dual-specificity phosphatase 1
HADb: Human autophagy database
HSPA5: Heat shock protein family A (Hsp70) member 5
HE: Hematoxylin and eosin
HSPA8: Heat shock protein family A (Hsp70) member 8
HSPB1: Heat shock protein family B (small) member 1
MSigDB: Molecular signatures database
mTOR: Mammalian target of rapamycin
mTORC1: Mammalian target of rapamycin complex 1
NSAIDs: Clinical work, nonsteroidal anti-inflammatory drugs
PPI: Protein–protein interaction
PPP1R15A: Protein phosphatase 1 regulatory subunit 15A
TFs: Transcription factors
TGFβ: Transforming growth factor-β
