# Supplementary figures and images for: Screening for autophagy/hypoxia/ferroptosis/pyroptosis-related genes of tendon injury and repair in a rat model after celecoxib and lactoferrin treatment
Source: J Orthop Surg Res. 2023 May 25;18:383. doi: 10.1186/s13018-023-03856-9 (PMC10214725; doi:10.1186/s13018-023-03856-9)

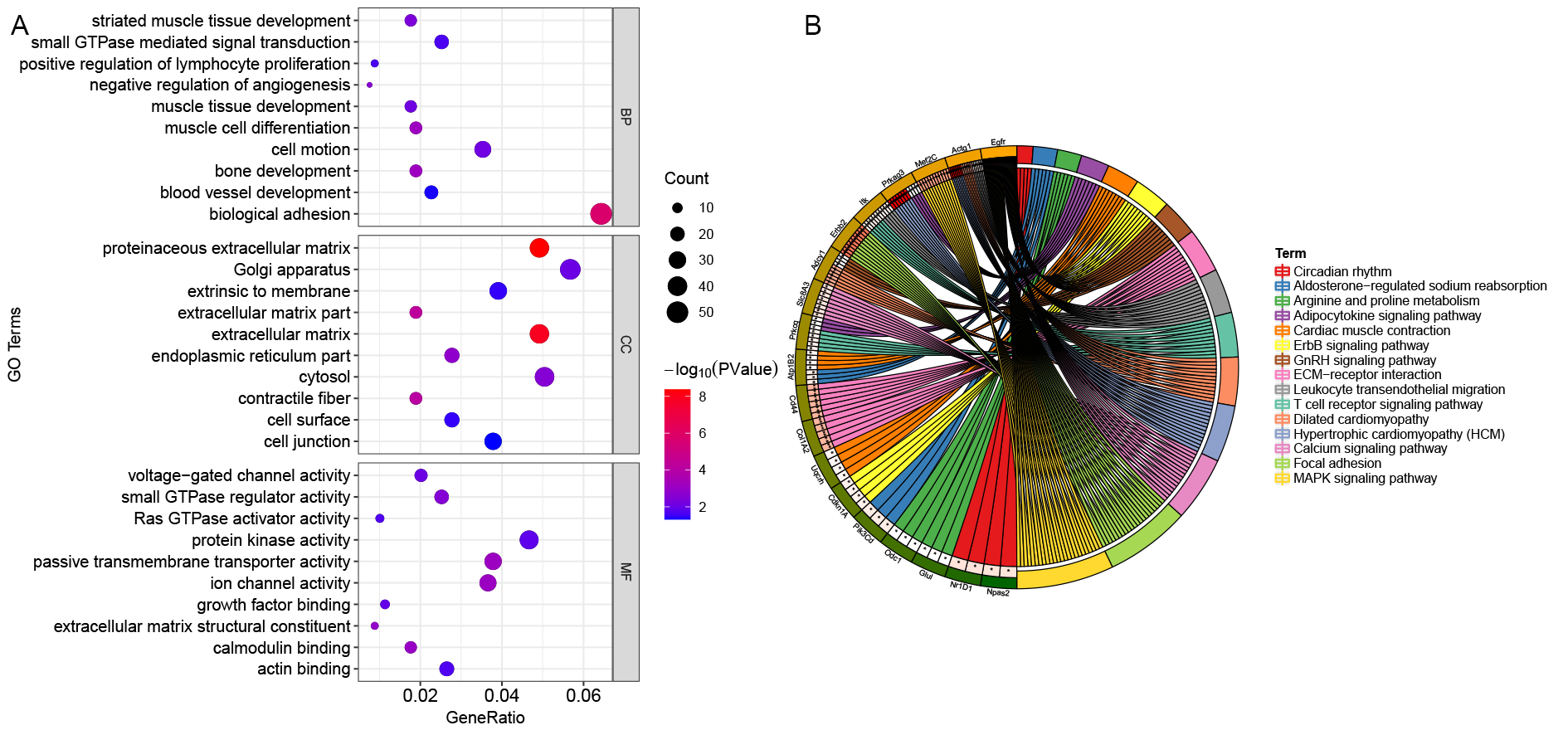

Supplement: Supplementary file 1 — Additional file 1. Figure S1 GO (A) and KEGG (B) enrichment analysis of DEmRNAs between injury model and celecoxib treatment group BP: biological process; CC: cytological component; MF: molecular function. [file 13018_2023_3856_MOESM1_ESM.tif]

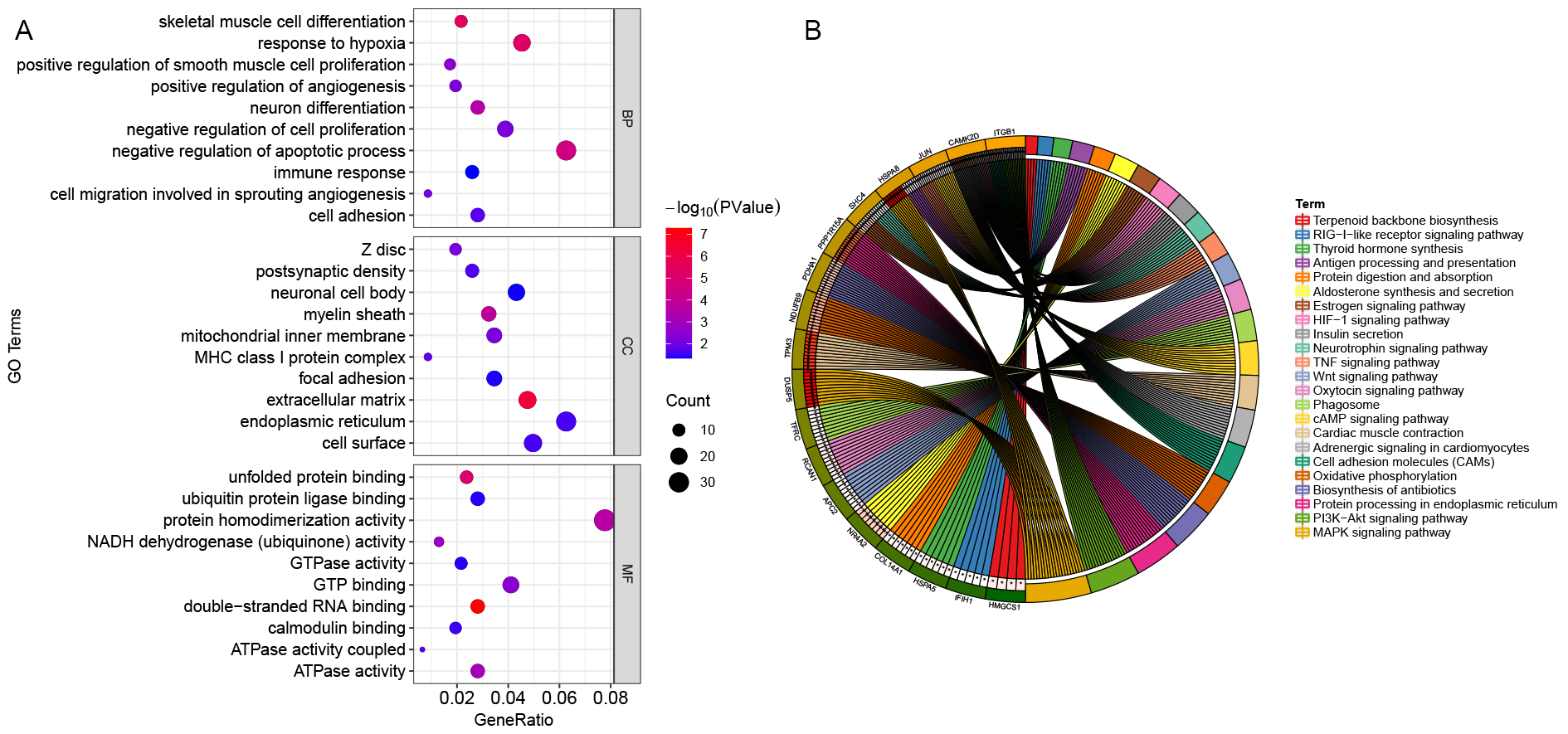

Supplement: Supplementary file 2 — Additional file 2. Figure S2 GO (A) and KEGG (B) enrichment analysis of DEmRNAs between injury model group and celecoxib + lactoferrin treatment group BP: biological process; CC: cytological component; MF: molecular function. [file 13018_2023_3856_MOESM2_ESM.tif]

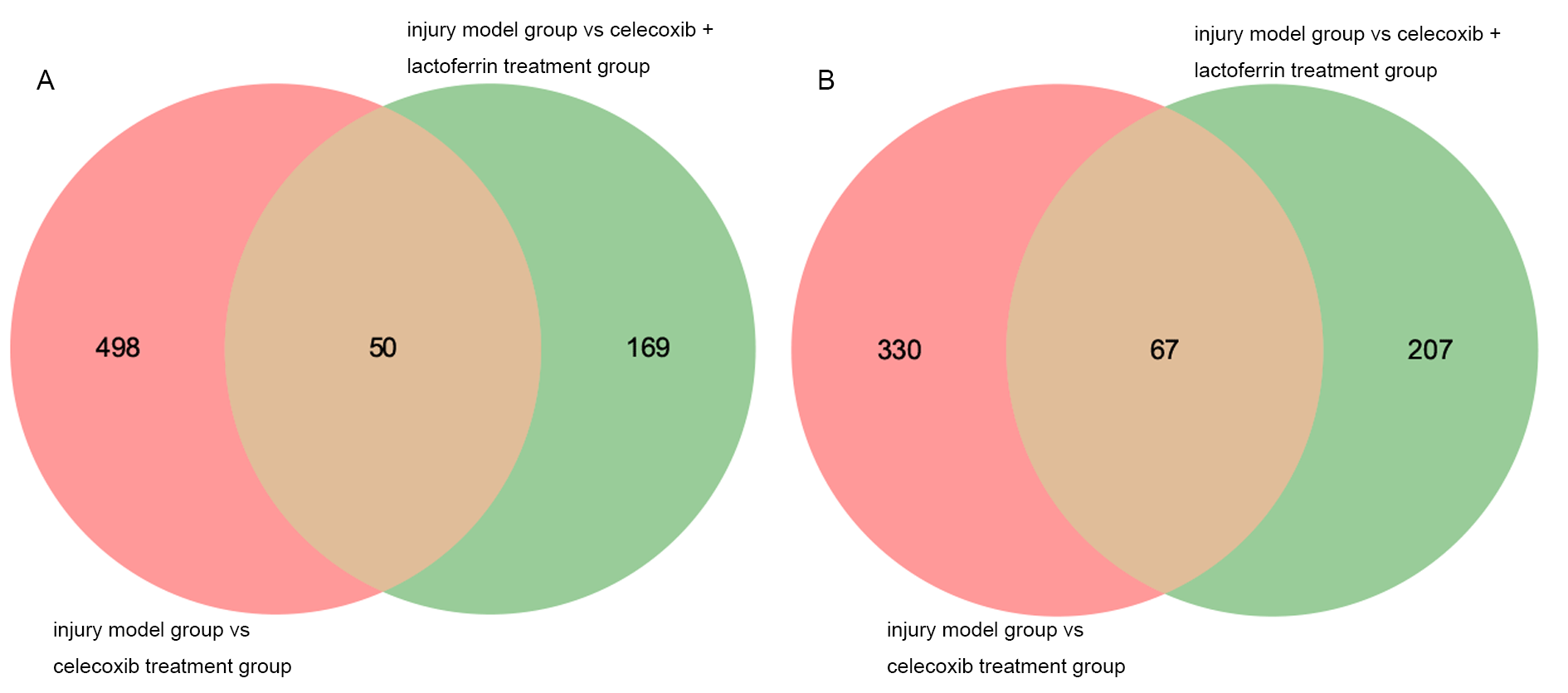

Supplement: Supplementary file 3 — Additional file 3. Figure S3 Identification of celecoxib + lactoferrin treatment group-specific DEmRNAs (A) Venn diagram of up-regulated DEmRNAs in injury model group vs celecoxib treatment group and injury model group vs celecoxib + lactoferrin treatment group. (B) Venn diagram of down-regulated DEmRNAs in injury model group vs celecoxib treatment group and injury model group vs celecoxib + lactoferrin treatment group. [file 13018_2023_3856_MOESM3_ESM.tif]
